# A Phosphatidylinositol 3‐Kinase Gamma Inhibitor Enhances Anti‐Programmed Death‐1/Programmed Death Ligand‐1 Antitumor Effects by Remodeling the Tumor Immune Microenvironment of Ovarian Cancer

**DOI:** 10.1002/mco2.70223

**Published:** 2025-07-23

**Authors:** Caixia Jiang, Rongyu Liu, Zhengyu Li

**Affiliations:** ^1^ Department of Obstetrics and Gynecology West China Second University Hospital, Sichuan University Chengdu P. R. China; ^2^ Key Laboratory of Obstetrics and Gynecologic and Pediatric Diseases and Birth Defects of Ministry of Education West China Second University Hospital, Sichuan University Chengdu P. R. China

**Keywords:** anti‐programmed cell death protein 1/programmed death ligand 1 | ovarian cancer | phosphatidylinositol 3‐kinase gamma inhibitor | tumor‐associated macrophages | tumor immune microenvironment

## Abstract

Previous studies have shown that the effectiveness of immune checkpoint blockade in the treatment of ovarian cancer (OC) is poor. A promising small molecule inhibitor targeting phosphatidylinositol 3‐kinase gamma (PI3Kγ) has recently been applied in combination with other drugs for tumor treatment. This study aimed to determine whether a PI3Kγ inhibitor can enhance the antitumor effects of anti‐programmed death‐1/programmed death ligand‐1 (anti‐PD‐1/PD‐L1) therapies in OC and to explore the underlying immunomolecular mechanism involved. Changes in the expression of PI3Kγ, PD‐1, PD‐L1, and tumor‐associated macrophages (TAMs) during the progression of OC were detected in clinical tissue samples. We also constructed a coculture system of OC cells with lymphocytes for in vitro study, and a subcutaneous and intraperitoneal implantation OC mouse model was constructed for in vivo studies. OC is an immunosuppressed tumor with predominant infiltration of M2 throughout the entire disease course. We also found that a PI3Kγ inhibitor combined with anti‐PD‐1 therapy can enhance the antitumor effects of anti‐PD‐1 agents by modulating the PI3Kγ‐AKT‐NF‐κB pathway, reprogramming TAMs, decreasing the number of myeloid‐derived suppressor cells, increasing the number of CD8^+^ T cells, and increasing the levels of proinflammatory factors; consequently, this approach transforms the tumor immune microenvironment of OC into a more active state.

## Graphical abstract

Phosphatidylinositol 3‐kinase gamma (PI3Kγ) inhibitor can sensitize the anti‐programmed death‐1/programmed death ligand‐1 (anti‐PD‐1/PD‐L1) response in ovarian cancer by modulating the PI3Kγ‐AKT‐NF‐κB pathway to remodel tumor immune microenvironment as an activated status, along with downregulating the expression of PD‐L1.

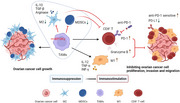

## 1 Introduction

The mortality rate of ovarian cancer (OC) is the highest among gynecological malignant tumors. It is estimated that there will be approximately 200,000 new cases of OC worldwide in 2024, resulting in nearly 130,000 deaths. Owing to late diagnosis, the 5‐year survival rate for most OC patients is only approximately 40% [1]. With the development of targeted drugs based on the molecular characteristics of OC, cytoreductive surgery combined with platinum‐based chemotherapy, with or without bevacizumab (a recombinant humanized monoclonal antibody targeting vascular endothelial growth factor), and, in some cases, followed by maintenance treatment with poly‐ADP‒ribose polymerase inhibitors (PARPis) (the first approved cancer drugs that specifically target the DNA damage response in BRCA1/2‐mutated OC), has emerged as a new model for the initial treatment of OC, which improves the survival outcome of patients who are BRCA mutation‐positive or HRD‐positive. However, treatment for OC that is BRCA wild‐type, HRD negative, recurrent, or refractory is still challenging [2, 3].

Immunotherapy, particularly immune checkpoint blockade (ICB), has recently contributed to significant advancements in the treatment of various malignant tumors, including melanoma and non‐small cell lung carcinoma. Concurrently, it has emerged as a promising research hotspot in the field of gynecological oncology. Beginning in 2021, pembrolizumab, an anti‐programmed death‐1 (PD‐1) inhibitor, has been recommended by the National Comprehensive Cancer Network guidelines for patients with OC exhibiting microsatellite instability‐high, mismatch repair‐deficient, or tumor mutational burden‐high tumors (≥10 mutations/megabase) who have no satisfactory alternative treatment options [4]. However, the published data on the use of anti‐PD‐1/programmed death ligand‐1 (anti‐PD‐1/PD‐L1) monotherapy in unscreened OC patients demonstrates low efficacy, with a response rate of only approximately 8%–15% [5, 6]. Therefore, improving the efficacy of anti‐PD‐1/PD‐L1 therapy for OC requires an in‐depth investigation of the underlying factors affecting the response or resistance to tumor immune checkpoint inhibitors.

OC is an “immunosuppressive tumor” or “cold tumor” characterized by a low tumor mutational burden, low tumor‐infiltrating lymphocytes (TILs), and low PD‐L1 expression. The poor immune response rate is typically closely related to the suppressed nature of the tumor immune microenvironment (TIME) in OC due to the high infiltration of immunosuppressive cell populations, such as tumor‐associated macrophages (TAMs), myeloid‐derived suppressor cells (MDSCs), and regulatory T cells (Tregs), as well as high levels of immunosuppressive cytokines [7, 8]. TAMs are the main cellular components of the solid tumor TIME and are highly plastic, and their phenotype can be transformed under different induced stimulus conditions. M1 macrophages have pro‐inflammatory and tumor cell‐killing functions, whereas M2 macrophages can inhibit CD8^+^ T‐cell activation and promote Treg recruitment through soluble suppressors, thus contributing to the development of tumor immune escape [9, 10, 11].

Phosphatidylinositol 3‐kinase gamma (PI3Kγ) is a heterodimer encoded by the phosphatidylinositol‐4,5‐bisphosphate 3‐kinase catalytic subunit gamma (PIK3CG) gene, which is selectively expressed in leukocytes, particularly within myeloid cells. PI3Kγ promotes the activation of C/EBPβ in a manner dependent on the mammalian target of rapamycin (mTOR) while inhibiting NF‐κB signaling, resulting in the transcription of immunosuppressive factors such as transforming growth factor‐β1 (TGF‐β1) and interleukin (IL)‐10. The application of a PI3Kγ inhibitor can reverse these effects. Eganelisib, a potent and selective small‐molecule PI3Kγ inhibitor, has been developed as a TAM‐reprogramming cancer immunotherapy with the potential to treat a broad range of cancers [12, 13]. Two preclinical studies published in *Nature* revealed that PI3Kγ acts as a molecular switch that regulates the immune functions of TAMs, thereby remodeling the TIME of melanoma and breast cancer models. The PI3Kγ inhibitor reprograms TAMs from an immune‐suppressive phenotype to an immune‐activating phenotype, enhancing the activity of ICB therapies (e.g., anti‐PD‐1 and anti‐cytotoxic T‐lymphocyte‐associated protein 4 [CTLA‐4] antibodies). Concurrently, it reduces the infiltration of MDSCs and promotes the activation of effector T cells, increasing their cytotoxic effects and further remodeling the TIME [14, 15, 16]. Furthermore, recent clinical trials have demonstrated that the addition of a PI3Kγ inhibitor can overcome resistance to ICB in patients with triple‐negative breast cancer (myeloid‐rich ICB‐refractory tumors) regardless of PD‐L1 status, and it has been shown to be safe for use in patients with multiple advanced solid tumors [17, 18]. The Cancer Genome Atlas genome analysis revealed that the expression of PIK3CG is significantly elevated in OC tissues. However, there is a notable lack of research on the application of PI3Kγ inhibitors in the context of anti‐PD‐1/PD‐L1 therapy for OC.

In summary, the present study aimed to determine whether a PI3Kγ inhibitor can enhance the antitumor effects of anti‐PD‐1/PD‐L1 therapy in OC. Additionally, we preliminarily explored the immune molecular mechanisms involved in the remodeling of the TIME by PI3Kγ in sensitizing the anti‐PD‐1/PD‐L1 therapeutic response.

## 2 Results

### 2.1 Expression, Prognosis, Functional Analysis, and Mutational Characterization of PIK3CG

**FIGURE 1:**
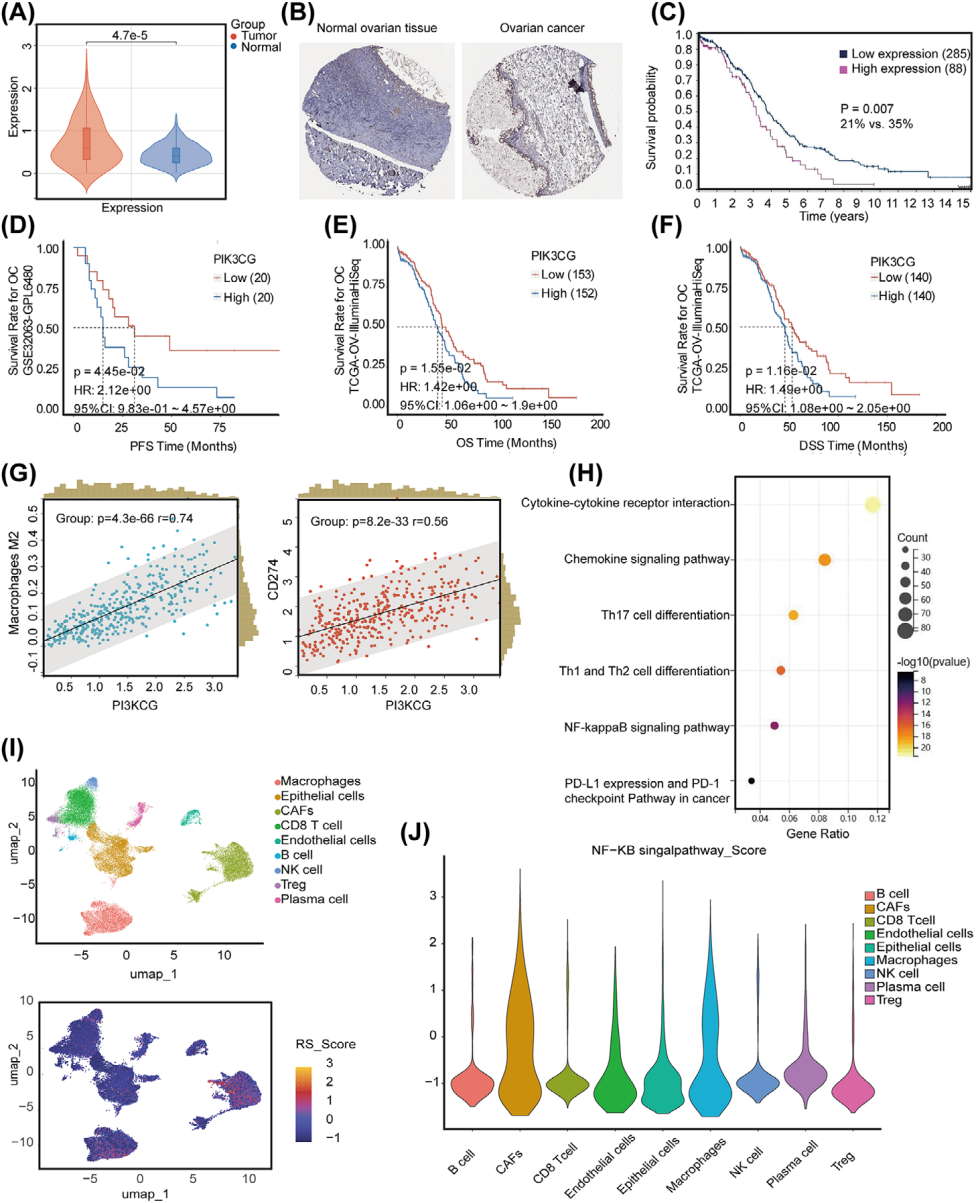
Expression, prognosis, functional analysis, and mutational characterization of PIK3CG. **(A, B)** Expression of PIK3CG in OC and normal ovarian tissue. **(C–F)** The expression of PIK3CG was associated with poor prognosis in OC patients (TCGA‐OV and GSE32062, (**C**) PFS, (**D**) OS, (**E**) DSS. HPA database, and (**F**) 5‐year OS). (**G**) CIBERSORT: PIK3CG was positively correlated with both M2 macrophages and PD‐L1. (**H**) KEGG and Hallmark: High expression of PIK3CG gene involved NF‐κB signaling pathway. (**I**) GSE154600 database: PIK3CG expression was elevated in macrophages within the TIME of OC. (**J**) NF‐κB signaling pathway had higher scores in macrophages. OC: ovarian cancer, PFS: progression‐free survival, OS: overall survival, DSS: disease‐specific survival.

An analysis of data from the TCGA‐OV, GTEx, HPA, and GSE32062 databases revealed that the expression of PIK3CG in OC tissue was significantly greater than that in normal ovarian tissue (*p* < 0.01) (Figure 1A, B). Kaplan‐Meier curves stratified by median PIK3CG level revealed that patients with higher PIK3CG expression had significantly shorter progression‐free survival (PFS), overall survival (OS), and disease‐specific survival (DSS) than those with lower PIK3CG expression (*p* < 0.05) (Figure 1C–F). We used the CIBERSORT algorithm for immune infiltration analysis and detected positive correlations between PIK3CG and both M2 macrophages (*r* = 0.74, *p* < 0.01) and PD‐L1 (*r* = 0.56, *p* < 0.01) (Figure 1G). High expression levels of PIK3CG and cytokine‒cytokine receptor interactions, as well as activation of the NF‐κB signaling pathway and PD‐L1 expression, were strongly correlated, according to KEGG and hallmark pathway analyses (Figure 1H). Given the degree of their correlation and their functional roles, these pathways were selected for further experiments. Furthermore, on the basis of the GSE154600 dataset, SingleR functions in conjunction with cell markers were utilized for cell annotation, revealing elevated expression of PIK3CG in macrophages within the TIME of OC (Figure 1I). Additionally, analysis using the addmodules core function indicated a higher score for the NF‐κB signaling pathway in macrophages and cancer‐associated fibroblasts (CAFs) (Figure 1J).

### 2.2 Changes in the Expression of PI3Kγ, PD‐1, PD‐L1, and TAMs During the Progression of OC

**FIGURE 2:**
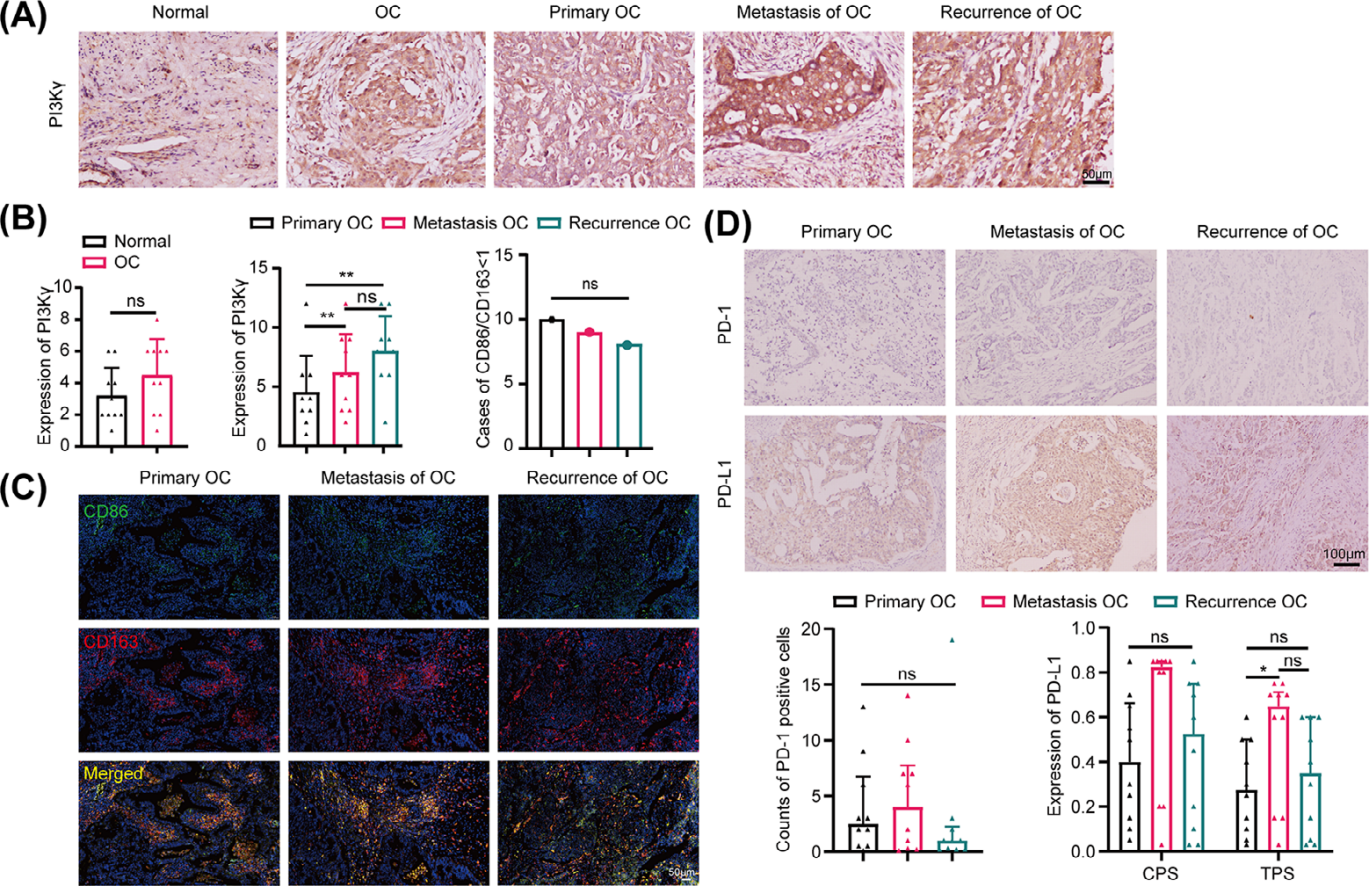
Changes in the expression of PI3Kγ, PD‐1, PD‐L1, and TAMs during the progression of OC. **(A, B)** Expression of PI3Kγ in normal ovarian tissue, OC, and the primary‐metastasis‐recurrence progression of HGSOC (n = 10). 200×; Scale bar: 50 µm. **(B, C)** Expression of M1 (CD68+CD86+) and M2 (CD68+CD163+) in the primary‐metastasis‐recurrence progression of HGSOC (n = 10). 200×; Scale bar: 50 µm. **(D)** Expression of PD‐1 and PD‐L1 in the primary‐metastasis‐recurrence progression of HGSOC (n = 10). TAMs: tumor‐associated macrophages, OC: ovarian cancer, HGSOC: high‐grade serous OC. CPS: combined positive score, TPS: tumor cell proportion score. 100×; Scale bar: 100 µm. Median (Quartile). ns, not significant; *p < 0.05, **p < 0.01.

A total of 10 paired paraffin‐embedded primary‐metastatic‐recurrent lesions and 10 paired lesions of healthy ovaries (stage I) were collected from patients with high‐grade serous OC (HGSOC) surgery, and the detailed clinical data are shown in Table S1. According to the immunohistochemistry (IHC) results, PI3Kγ expression trended higher in OC than in normal ovarian tissues (4.50 ± 2.00 vs. 3.2 ± 1.75, *p =* 0.152), was greater in metastatic (5.75 ± 3.26, *p* < 0.001) and recurrent (7.88 ± 2.94, *p* < 0.001) lesions than in primary lesions (4.56 ± 3.24), and trended upwards in recurrent lesions compared to metastatic lesions (*p* = 0.401), although some of the differences were not statistically significant (Figure 2A,B). Besides, M1 (CD68+CD86+) and M2 (CD68+CD163+) macrophages exhibited only a small amount of infiltration throughout the development of HGSOC. We conducted triple immunofluorescence staining, and the results indicated that the M1/M2 ratio was ≤1 in the vast majority of sections from primary OC lesions, metastatic OC lesions, and recurrent OC lesions, and there was no difference among the three groups (Figure 2B,C). In addition, PD‐1 expression was low in all the primary‐metastatic‐recurrent HGSOC lesions, and PD‐L1 expression tended to increase in the metastatic and recurrent lesions compared with the primary lesions, although most of the differences were not statistically significant (Figure 2D).

### 2.3 Induced Differentiation and Identification of Peripheral Blood Mononuclear Cells/Mouse Spleen Lymphocytes

**FIGURE 3:**
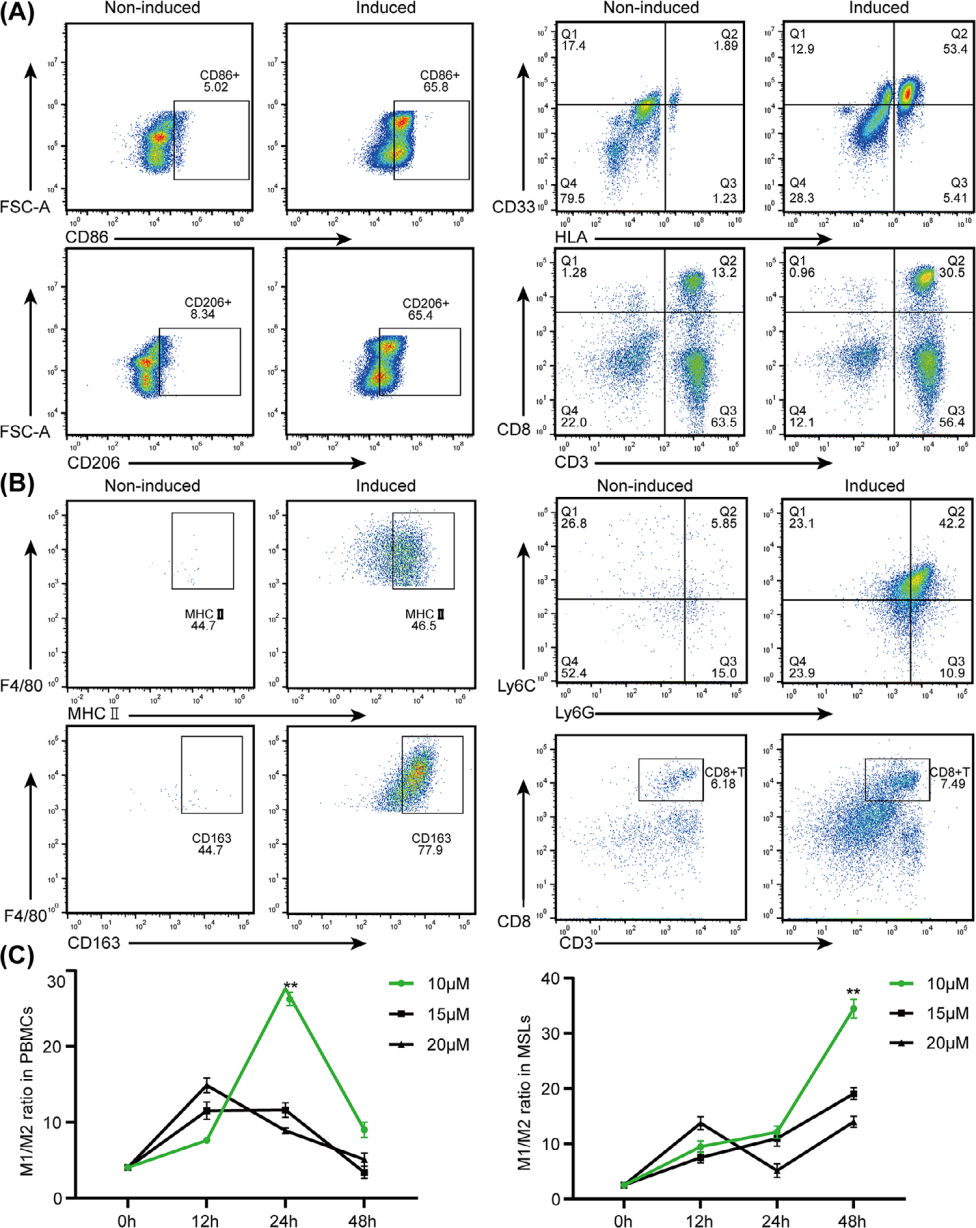
Changes in subpopulations after induction by M‐CSF, IL‐4, and IL‐13 of PBMCs and MSLs. **(A)** The proportions of M1 (CD11b+CD86+), M2 (CD11b+CD206+), MDSCs (CD11b+HLA‐CD33+), and CD8^+^ T cells (CD3+CD8+) in PBMCs. **(B)** The proportions of M1 (CD11b+F4/80+MHCII+), M2 (CD11b+F4/80+CD163+), MDSCs (CD11b+Ly6G+Ly6C‐) and CD8^+^ T cells (CD3+CD8+) in MSLs. **(C)** Changes of M1/M2 ratio in PBMCs and MSLs treated with IPI‐549 at concentrations of 10, 15, and 20 µM for 12, 24, and 48 h. PBMCs: peripheral blood mononuclear cells, MSLs: mouse spleen lymphocytes. *p < 0.05; **p < 0.01.

After induction by drug stimulation, flow cytometry (FCM) analysis revealed a significant increase in the number of mature macrophages, a decrease in the proportion of MDSCs, and an almost unchanged proportion of CD8^+^ T cells after induction compared with those in peripheral blood mononuclear cells (PBMCs)/mouse spleen lymphocytes (MSLs) without induction (Figure 3A, B). The highest M1/M2 ratio was determined by FCM analysis after IPI‐549 was administered to PBMCs (10 µM × 24 h) and MSLs (10 µM×48 h) (Figure 3C).

### 2.4 IPI‐549 Enhances the Ability of Anti‐PD‐1 to Inhibit Proliferation, Migration, and Invasion and Promote the Apoptosis of OC Cells

**FIGURE 4:**
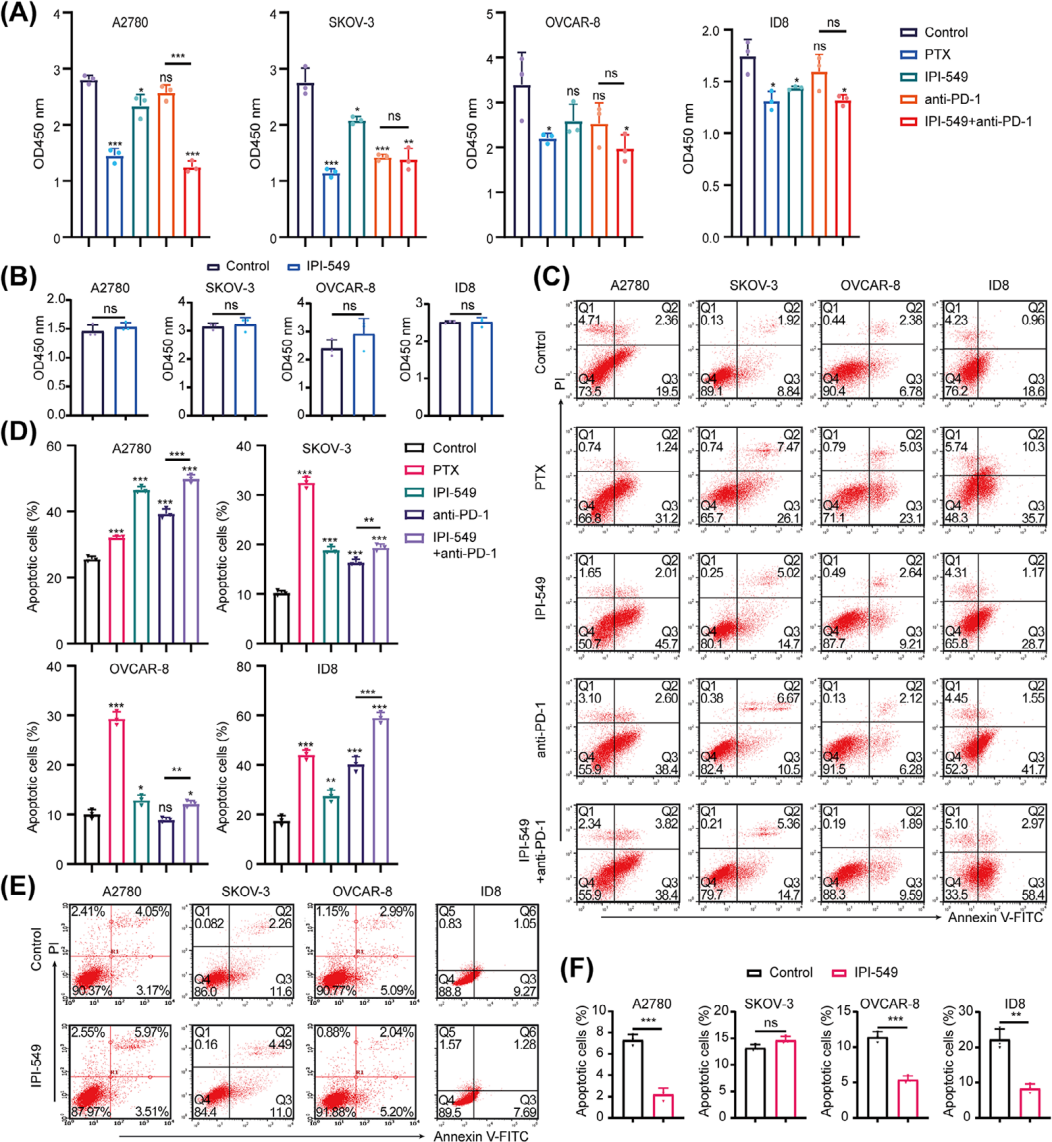
**(A)** Effects of IPI‐549 and anti‐PD‐1 agents alone or in combination on the proliferation viability of OC cells under the cell co‐culture system. **(B)** Effect of IPI‐549 alone on the proliferation viability of OC cells that were not co‐cultured with PBMCs or MSLs. *p < 0.05, **p < 0.01, ***p < 0.001. **(C, D)** Effects of IPI‐549 and anti‐PD‐1 agents alone or in combination on the pro‐apoptosis effect of OC cells under the cell co‐culture system. **(E, F)** Effects of IPI‐549 alone on the pro‐apoptosis effect of OC cells that were not co‐cultured with PBMCs or MSLs. OC: ovarian cancer, PBMCs: peripheral blood mononuclear cells, MSLs: mouse spleen lymphocytes. ns, not significant; *p < 0.05; **p < 0.01.

**FIGURE 5:**
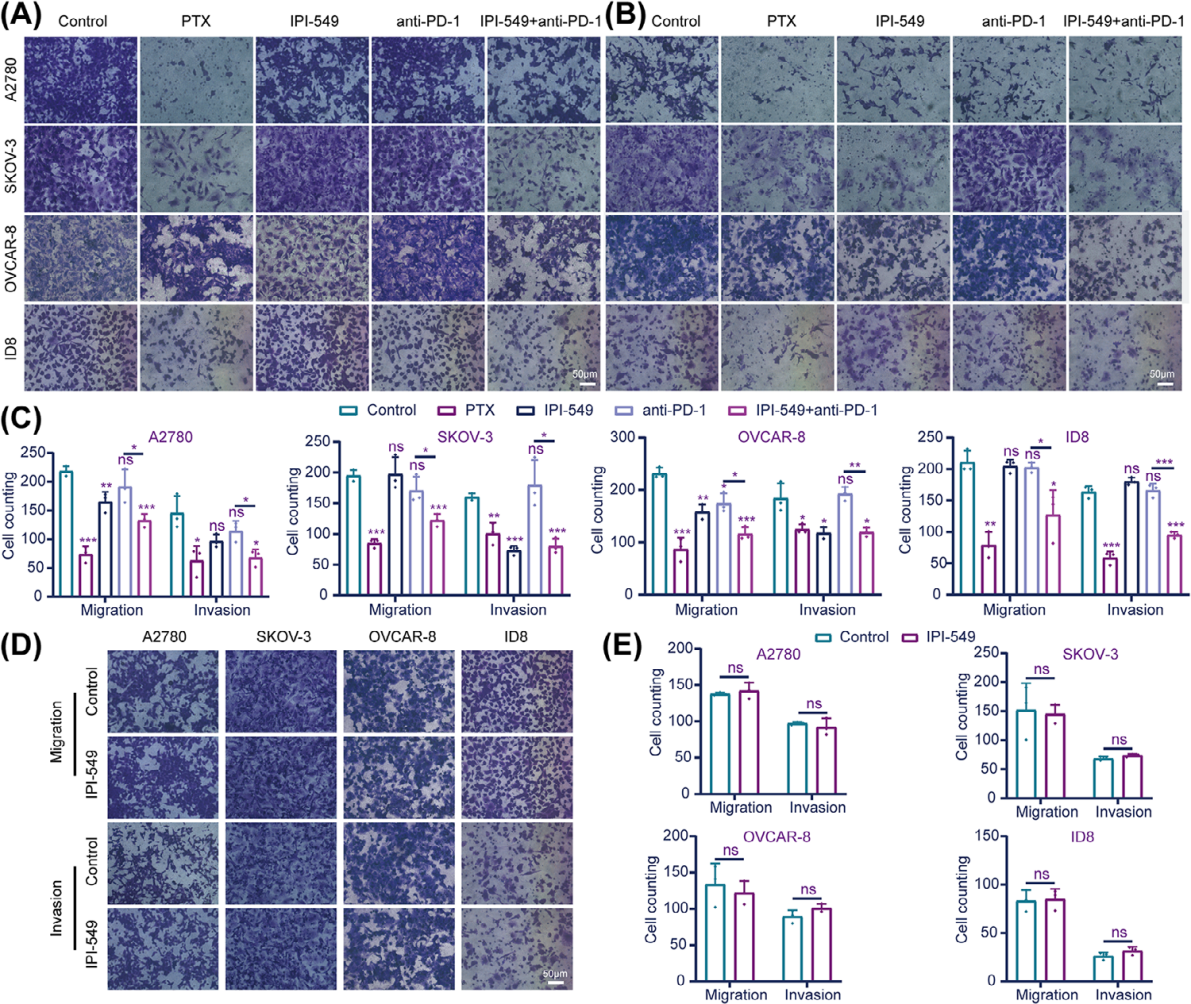
Effects of IPI‐549 and anti‐PD‐1 agents alone or in combination on the **(A, C)** migration and **(B, C)** invasion ability of OC cells under the cell co‐culture system. **(D, E)** Effects of IPI‐549 alone on the migration and invasion ability of OC cells that were not co‐cultured. OC: ovarian cancer. 200×; Scale bar: 50 µm. ns, not significant; *p < 0.05; **p < 0.01; ***p < 0.001.

To explore the effects of IPI‐549 and anti‐PD‐1 alone or in combination on OC cells in the cell coculture system, the proliferation, viability, apoptosis, migration, and invasion capacity of five groups of OC cells (A2780, SKOV‐3, OVCAR‐8, and ID8) in the upper chamber were measured. A Cell Counting Kit‐8 assay revealed that, compared with the control group, both the IPI‐549 (*p* < 0.05 for A2780, SKOV‐3 and ID8) and anti‐PD‐1 groups (*p* < 0.05 for SKOV‐3) significantly inhibited the proliferation of some OC cell lines, whereas the IPI‐549+anti‐PD‐1 combination inhibited the proliferation of all OC cell lines (*p* < 0.05) and exceeded the ability of anti‐PD‐1 alone to inhibit their proliferation (*p* < 0.001 for A2780). In addition, the results also demonstrated that IPI‐549 alone had no significant effect on the proliferation of OC cell lines that were not cocultured with PBMCs or MSLs (*p* > 0.05) (Figure 4A,B). The findings also demonstrated that paclitaxel (PTX) increased the degree of apoptosis in all OC cell lines, whereas the combination of IPI‐549, anti‐PD‐1, and IPI‐549+anti‐PD‐1 increased the number of OC cell lines compared with that in the control group. However, the two drugs combined were more effective at promoting apoptosis than the anti‐PD‐1 agent alone (*p* < 0.01) (Figure 4C,D). IPI‐549 did not have any proapoptotic effects on OC cell lines that were not cocultured with PBMCs or MSLs (*p* > 0.05), which is notable (Figure 4E,F). The Transwell results demonstrated that IPI‐549 and anti‐PD‐1 were only able to partially inhibit the ability of OC cell lines to migrate or invade when compared to the control group, whereas the combination of PTX and IPI‐549+anti‐PD‐1 prevented the migration and invasion of all OC cell lines (*p* < 0.05). Furthermore, the combination of the two drugs had a greater ability to inhibit migration and invasion than did anti‐PD‐1 alone (*p* < 0.05) (Figure 5A–C). Additionally, the results revealed that IPI‐549 alone had no significant effect on the ability of OC cell lines that were not cocultured with PBMCs or MSLs to migrate and invade (*p* > 0.05) (Figure 5D,E).

### 2.5 IPI‐549 Remodels the TIME of OC in the Cell Coculture System

**FIGURE 6:**
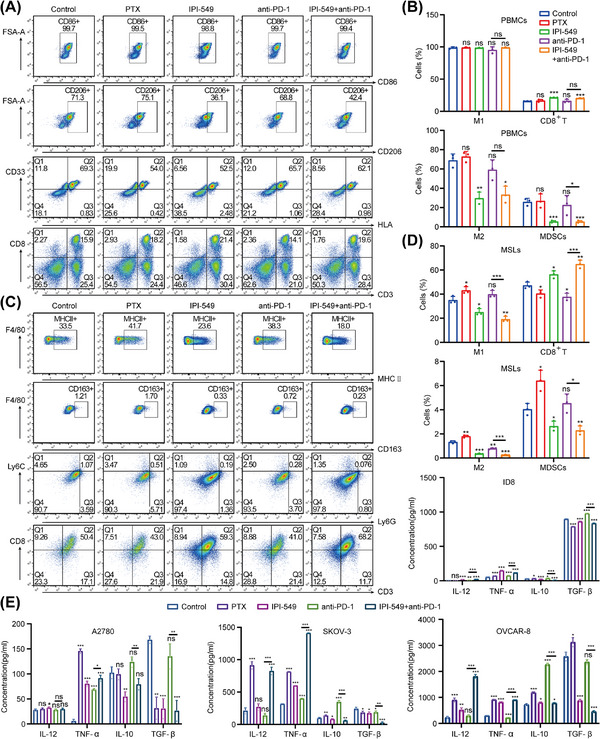
IPI‐549 and anti‐PD‐1 agents alone or in combination on the percentages of immune cell subpopulations and inflammatory cytokines in **(A, B)** PBMCs and **(C, D)** MSLs under the cell co‐culture system. **(A)** PBMCs: M1 (CD11b+CD86+), M2 (CD11b+CD206+), MDSCs (CD11b+HLA‐CD33+), and CD8^+^ T cells (CD3+CD8+). **(C)** MSLs: M1 (CD11b+F4/80+MHCII+), M2 (CD11b+F4/80+CD163+), MDSCs (CD11b+Ly6G+Ly6C‐) and CD8^+^ T cells (CD3+CD8+). **(E)** Proinflammatory cytokines (IL‐12, TNF‐α); anti‐inflammatory cytokines (IL‐10, TGF‐β). PBMCs: peripheral blood mononuclear cells, MSLs: mouse spleen lymphocytes. ns, not significant; *p < 0.05; **p < 0.01; ***p < 0.001.

The changes in the proportions of M1, M2, MDSCs, and CD8^+^ T cells in PBMCs and MSLs were detected by FCM (Figure 6A–D). Compared with that in the control group, the proportion of M1 macrophages in PBMCs in the drug treatment group did not change (*p* > 0.05), whereas the proportion of M2 macrophages in the IPI‐549 group and the IPI‐549+anti‐PD‐1 group decreased significantly (*p* < 0.001); as a result, the M1/M2 ratio tended to increase in these two groups (*p* > 0.05). The M1/M2 ratio was also significantly increased in the IPI‐549 and IPI‐549+anti‐PD‐1 groups in MSLs (*p* < 0.01). More importantly, the M1/M2 ratio tended to increase in the IPI‐549+anti‐PD‐1 group compared with those in the anti‐PD‐1 group in both PBMCs (*p* > 0.05) and MSLs (*p* < 0.05). Compared with those in the control group, both the IPI‐549 group and the IPI‐549+anti‐PD‐1 group presented a decrease in the proportion of MDSCs and an increase in the number of CD8^+^ T cells among the PBMCs (*p* < 0.001) and MSLs (*p* < 0.05), whereas there were no significant changes in most of the PTX and anti‐PD‐1 groups (*p* > 0.05). Moreover, the percentage of MDSCs decreased while the percentage of CD8^+^ T cells increased in the IPI‐549+anti‐PD‐1 group and showed a decreasing trend compared with the anti‐PD‐1 group for both PBMCs and MSLs (most Groups *p* < 0.05). Additionally, the levels of proinflammatory cytokines such as IL‐12 and TNF‐α and anti‐inflammatory cytokines such as IL‐10 and TGF‐β in the supernatants of the five groups showed corresponding changes (Figure 6E).

### 2.6 IPI‐549 Enhances the Inhibitory Effect of Anti‐PD‐1 Therapy on the Growth of Subcutaneous and Intraperitoneal OC in Mice

**FIGURE 7:**
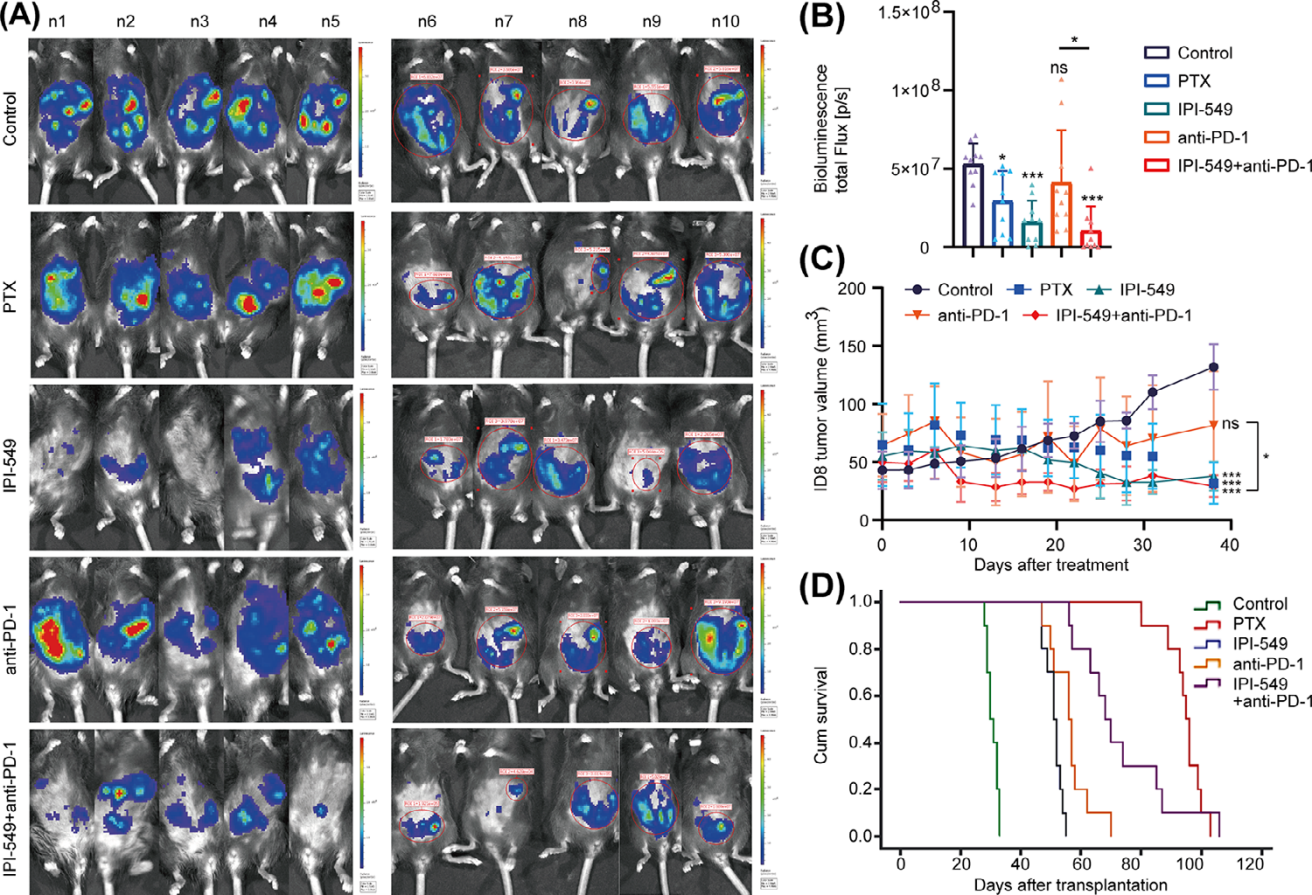
IPI‐549 enhanced the inhibitory effect of the anti‐PD‐1 agent on the growth of subcutaneous and intraperitoneal OC in mice. **(A, B)** In vivo imaging of intraperitoneal OC in mice (n = 10). **(C)** Tumor volume (n = 5). **(D)** The survival time of mice (n = 10). OC: ovarian cancer. ns, not significant; *p < 0.05; ***p < 0.001.

In vivo imaging of intraperitoneal OC in mice revealed that the IPI‐549 (*p* < 0.001), IPI‐549+anti‐PD‐1 (*p* < 0.001), and PTX (*p* = 0.010) groups presented significantly lower tumor burdens than the control group did, whereas the anti‐PD‐1 group (*p* = 0.318) presented no significant changes in tumor burden. Moreover, mice in the IPI‐549 + anti‐PD‐1 group had a lower tumor burden than those in the anti‐PD‐1 group (*p* = 0.016) (Figure 7A,B). The results revealed that the tumor volume of the mice in the PTX group (31.95 ± 17.70 mm^3^, *p* < 0.001), IPI‐549 group (37.88 ± 12.59 mm^3^, *p* < 0.001), and IPI‐549+anti‐PD‐1 group (29.17 ± 9.38 mm^3^, *p* < 0.001) was significantly lower than that of the control group (131.93 ± 19.47 mm^3^), whereas the tumor volume of the mice in the anti‐PD‐1 group (81.69 ± 46.64 mm^3^, *p* = 0.105) also decreased, but the difference was not statistically significant. The tumor volume of the mice in the IPI‐549+anti‐PD‐1 combination group also decreased compared with that of the anti‐PD‐1 group (*p* = 0.042) (Figure 7C). Moreover, the mice were administered the drug for one month. Our results indicated that the mice in the PTX group (median 95 days, *p* = 5 × 10^−5^), IPI‐549 group (median 51 days, *p* = 5 × 10^−5^), anti‐PD‐1 group (median 56 days, *p* = 5 × 10^−5^), and IPI‐549 + anti‐PD‐1 group (median 68 days, *p* = 5 × 10^−5^) had longer median survival times than did the mice in the control group (median time 30 days). Compared with the anti‐PD‐1 group, the IPI‐549 + anti‐PD‐1 group had a longer median survival time (*p* = 0.003) (Figure 7D). In addition, the in vivo administration of IPI‐549 had no significant effect on body weight (*p* > 0.05) and no significant toxicity to any of the organs of the mice (Figure S2A,B).

### 2.7 IPI‐549 remodels the TIME of OC by regulating the PI3Kγ‐AKT‐NF‐κB pathway in mice

**FIGURE 8:**
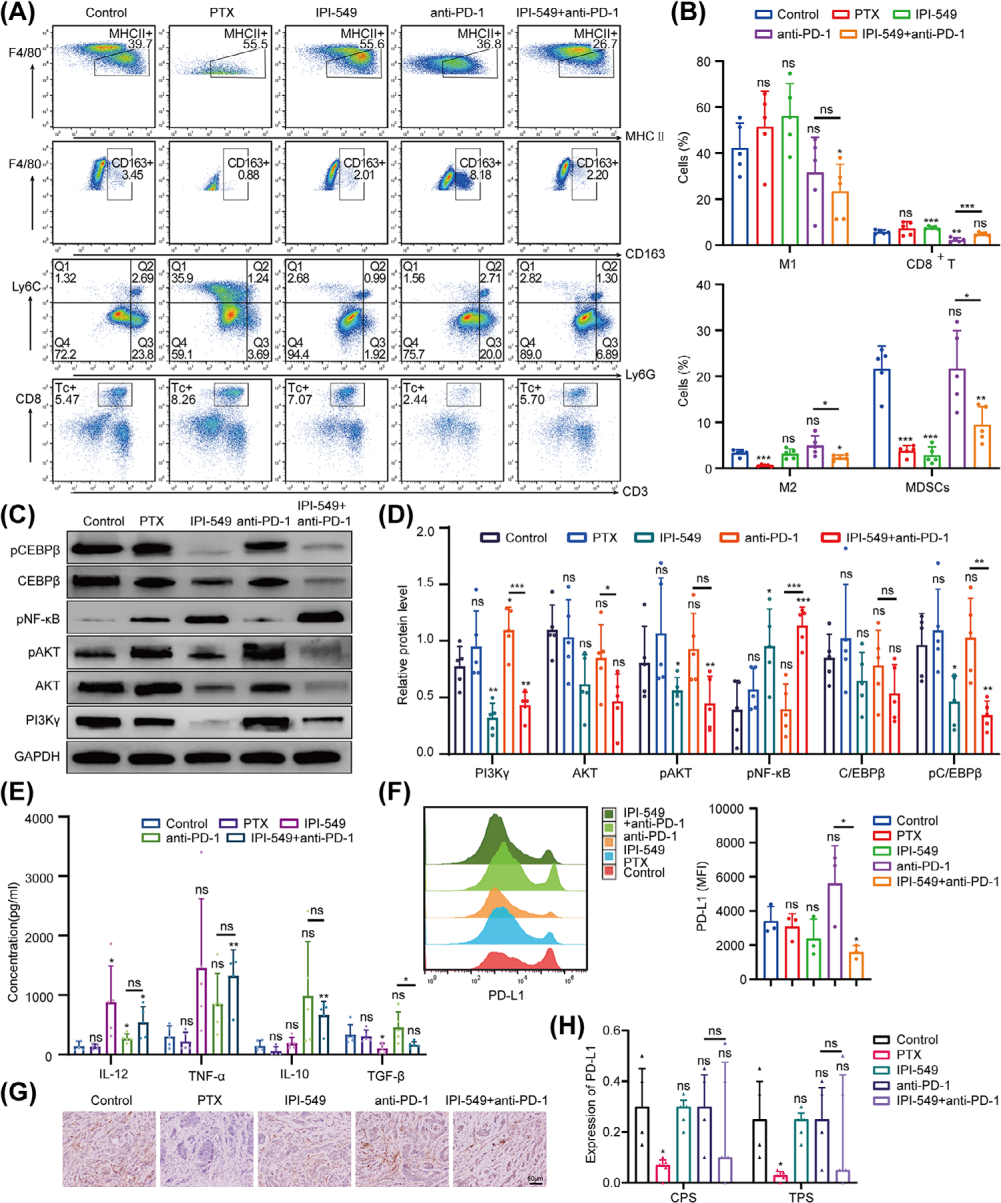
**(A, B)** Effects of IPI‐549 and anti‐PD‐1 agents alone or in combination on the ratio of M1, M2, MDSCs, and CD8^+^ T cells in exfoliated cells from the mice peritoneal cavity. M1 (CD11b+F4/80+MHCII+), M2 (CD11b+F4/80+CD163+), MDSCs (CD11b+Ly6G+Ly6C‐) and Tc (CD8^+^ T cells, CD3+CD8+). **(C, D)** IPI‐549 remodeled TIME of subcutaneous OC in mice: regulation of PI3Kγ‐AKT‐NF‐κB pathway‐associated target proteins. **(E)** Effects of IPI‐549 and anti‐PD‐1 agents alone or in combination on the proinflammatory (IL‐12, TNF‐α) and anti‐inflammatory (IL‐10, TGF‐β) cytokines in mice peritoneal lavage fluid. IPI‐549 downregulated the expression of PD‐L1 in OC of mice. **(F)** The expression of PD‐L1 in intraperitoneal OC of mice. **(G, H)** The expression of PD‐L1 in subcutaneous OC of mice. OC: ovarian cancer, TIME: tumor immune microenvironment, CPS: combined positive score, TPS: tumor cell proportion score. 200×; Scale bar: 50 µm. Median (Quartile). ns, not significant; *p < 0.05; **p < 0.01; ***p < 0.001.

We next investigated the potential mechanism by which IPI‐549 remodels the TIME of OC in mice. Briefly, the peritoneal cavity M1/M2 ratios of the mice in the IPI‐549 (*p* = 0.085) groups were greater than those in the control group, and moreover, there was an increasing trend in the IPI‐549+anti‐PD‐1 group (*p* = 0.575) compared with those in the anti‐PD‐1 group, although the difference was not statistically significant. In addition, compared with those in the control group, the number of MDSCs were lower in the IPI‐549 group (*p* < 0.001) and IPI‐549+anti‐PD‐1 group (*p* = 0.003). Compared with the anti‐PD‐1 group, the IPI‐549+anti‐PD‐1 group presented a decrease in the proportion of MDSCs (*p* = 0.018) and a significant increase in the number of CD8^+^ T cells (*p* < 0.001) (Figure 8A,B). Besides, the Western blot results revealed that compared with those in the control group, PI3Kγ, pAKT, and pCEBPβ protein levels were lower in the IPI‐549 group and IPI‐549+anti‐PD‐1 group in subcutaneous OC mice (*p* < 0.01 or *p* < 0.05), whereas NF‐κB protein phosphorylation levels were increased (*p* < 0.01). In addition, these proteins were not significantly changed in the PTX group or the anti‐PD‐1 group (*p* > 0.05) (Figure 8C,D). In addition, similar to the results of the in vitro cell experiments, the peritoneal lavage fluid of the mice revealed that the IL‐12 and TNF‐α levels in the IPI‐549+anti‐PD‐1 group tended to increase overall compared with those in the control group and anti‐PD‐1 group, whereas the IL‐10 and TGF‐β levels tended to decrease overall (some Groups *p* < 0.05) (Figure 8E).

### 2.8 IPI‐549 downregulates the expression of PD‐L1 in OC in mice

Analysis of PD‐L1 expression in exfoliated cells from the peritoneal cavity of mice showed that the median fluorescence intensity (MFI) of PD‐L1 in the IPI‐549+anti‐PD‐1 group was lower than that in the control group (*p* < 0.05), whereas there was an increasing trend in the anti‐PD‐1 group (*p* = 0.181), although the difference was not statistically significant (Figure 8F). Furthermore, compared to the control group, the median PD‐L1 scores were lower in both the IPI‐549 group and the IPI‐549+anti‐PD‐1 group in subcutaneous OC mice; however, these differences were not statistically significant (*p* > 0.05), and no changes were observed in the anti‐PD‐1 group (Figure 8G, H).

## 3 Discussion

Clinical trials have shown that ICB is less effective in the treatment of OC than in other solid tumors, such as melanoma. In recent years, an increasing number of studies have explored the impact of TIME on ovarian oncogenesis and development to identify novel and effective immune targets [19, 20]. An increasing number of studies have shown that the TIME in OC is complexly heterogeneous and dynamically changes with disease progression and that multiple immune cells and their derivatives can mediate the switch between pro‐ and antitumor effects, which in turn influences tumor development and patient survival [21]. In this study, the paired HGSOC samples exhibited low M1 expression throughout the progression of HGSOC, whereas the infiltration abundance of M2 (compared with M1) was always dominant (although dichotomous methods such as M1 and M2 do not accurately reflect the functional complexity of this cellular phenotype) and was closely associated with the immunosuppressive state of OC, which is consistent with previous studies [22]. We also observed that low PD‐1 expression reflected the low infiltration of TILs in the microenvironment throughout the whole disease process. Previous studies have shown that PI3Kγ expression is increased in chronic granulocytic leukemia, invasive breast cancer and renal cancer and is closely involved in disease progression in T‐cell acute lymphoblastic leukaemia and pancreatic ductal adenocarcinoma [23]. PD‐L1 is one of the best biomarkers currently used to predict the tumor immune response and survival prognosis [8, 24]. In this study, bioinformatics analysis revealed that the expression of PIK3CG was abnormally upregulated in OC tissues in the TCGA‐OV, GTEx, HPA, and GSE32062 databases and that the expression of PIK3CG was associated with poor prognosis in OC patients. Our results were also consistent with those of the bioinformatics analysis; the expression of PI3Kγ was greater in OC than in normal ovarian tissues, and the expression of PI3Kγ increased with disease progression in HGSOC, suggesting that PI3Kγ is closely related to OC progression and prognosis and that the majority of current clinical trials of PD‐1/PD‐L1 inhibitors have been conducted in recurrent OC patients. Our bioinformatics analysis also revealed that PIK3CG was positively correlated with both M2 macrophages and PD‐L1 in OC, and the positive correlation between PIK3CG and M2 macrophages indicated that PIK3CG may be involved in the regulation of M1 and M2 abundance in OC and thus regulate immune activity. The positive correlation between PIK3CG and PD‐L1 suggests that PIK3CG may affect the anti‐PD‐1/PD‐L1 response rate by regulating the expression level of PD‐L1. These results were further verified by our in vivo and in vitro experiments.

To observe the interactions between OC cells and immune cell populations, as well as alterations in the biological properties and functions of both OC cells and immune cell populations, this study used previously established methods from the literature [25]. Specifically, 6‐well plates equipped with 0.4 µm Transwell chambers were utilized to create a noncontact cell coculture system. This innovative approach has not been previously documented in the literature for the investigation of PI3Kγ inhibitors. We tested in vitro antitumor effects by constructing a coculture system of four OC cell lines with PBMCs or MSLs. The combination of IPI‐549 enhanced anti‐proliferative effects, inhibited migration and invasion, and promoted the pro‐apoptotic antitumor effects of anti‐PD‐1 therapy on OC cells. Furthermore, the antitumor effects of the combination of these two drugs were also been confirmed in mice, and the median survival time of the mice in the combination group was longer than that of the mice in the anti‐PD‐1 monotherapy group. However, we also observed that IPI‐549 alone did not have antitumor effects on OC cells that were not co‐cultured in vitro. In contrast, IPI‐549 demonstrated antitumor effects on OC cells when they were co‐cultured with immune cell populations. This finding is consistent with previous studies reporting no effect of IPI‐549 on the proliferative viability of melanoma cells in vitro [16]. Therefore, how IPI‐549 enhances the antitumor effects of anti‐PD‐1 therapy by modulating the immune cell population in the coculture system and its immune mechanism need to be further investigated.

TAMs (mainly referred to as M2) can secrete suppressive cytokines and interact with other immune cells to form a suppressive TIME, which then becomes the key to tumor progression and immunotherapy resistance [20, 26]. MDSCs, the main drivers of intratumor local immunosuppression, gradually deplete the tumor microenvironment of nutrients essential for T lymphocyte function and produce reactive oxygen and immunosuppressive cytokines that directly suppress the organism's immunity [27]. In our study, we collected immune cells and cell supernatants from in vitro cocultured cell lines and collected abdominal exfoliated cells and liquid from an intraperitoneal OC mouse model for FCM analysis and enzyme‐linked immunosorbent assay (ELISA). The results demonstrated that IPI‐549 improved the antitumor effects by reprogramming TAMs (M2 cells repolarizing to M1 cells), decreasing MDSCs, increasing CD8^+^ T cells, increasing the levels of proinflammatory cytokines such as IL‐12 and TNF‐α, and reducing the levels of anti‐inflammatory cytokines such as IL‐10 and TGF‐β in both the cell coculture system and mice to remodel the TIME of OC, shifting immunosuppression to the immune response, and thus increasing the sensitivity of OC cells to anti‐PD‐1. Kaneda et al. [15] reported that a PI3Kγ inhibitor can synergize with anti‐PD‐1 to inhibit tumor proliferation and metastasis by remodeling the TIME in melanoma and breast cancer animal models. Moreover, they suggested that this antitumor effect was mediated by inactivating macrophages by knocking down PI3Kγ or through the use of PI3Kγ inhibitors. This process converts macrophages to the proinflammatory M1 phenotype, which indirectly increases the recruitment and cytotoxicity of CD8^+^ T cells and amplifies the Th1 response. This mechanism of reprogramming TAMs is similar to that in our study; however, we incorporated an additional model of in vitro cell coculture to provide further validation. In future research, we plan to integrate OC organoids or microfluidic chip models to further elucidate the subtle changes within the TIME. Liu et al. [28] reported that TG100‐115, a PI3Kγ inhibitor, targets TAMs via the PI3Kγ/AKT pathway. This process remodels the TIME and enhances the cytotoxic T‐cell‐mediated tumor immunotherapeutic effect, thereby overcoming resistance to anti‐PD‐1 therapy in residual tumor models of hepatocellular carcinoma following insufficient radiofrequency ablation. The function of PI3Kγ inhibitors in remodeling the TIME in our study has also been demonstrated in other studies involving immunosuppressive tumors, such as colon cancer and temozolomide‐resistant glioma [29, 30]. To date, a total of 36 studies have investigated the use of PI3Kγ inhibitors (IPI‐549 and TG100‐115) as a strategy to reverse myeloid‐driven immune suppression across 12 solid tumors. The combination of PI3Kγ inhibitors with various therapeutic modalities — including immune checkpoint inhibitors, chemotherapy, radiotherapy, biological agents, and vaccines — has shown increased antitumor effects, indicating a synergistic approach to overcoming immune suppression. However, there is a lack of experimental studies on this remodeling effect in OC [13]. It has also been reported that macrophage reprogramming from the M2 to the M1 phenotype may increase PD‐L1 expression, which in turn leads to the side effects of macrophages being in a “functional failure” state. The combination of PD‐L1/PD‐1 inhibitors could compensate for defects in macrophage reprogramming and provide a potential therapeutic strategy [31]. This finding provides further important supporting evidence for the combination of IPI‐549 and anti‐PD‐1 therapy in OC treatment in this study. Unexpectedly, in our in vivo experiments, IPI‐549 alone demonstrated an antitumor effect; however, this finding is preliminary and requires further validation. Previous in vivo research has similarly demonstrated the antitumor properties of IPI‐549 alone [16], which is consistent with our current results. The exact mechanisms underlying the antitumor effects of IPI‐549 alone will be investigated in our future studies.

Pathway‐related proteins were further examined to investigate the underlying molecular mechanism. Our bioinformatic analysis also revealed that high expression levels of PIK3CG in OC may play a role in the NF‐κB signaling pathway in TAMs. Our results demonstrated that IPI‐549 can inhibit AKT and CEBPβ phosphorylation and promote NF‐κB activation, indicating that IPI‐549 can remodel the TIME of OC by regulating the PI3Kγ‐AKT‐NF‐κB pathway. The tumor‐associated regulatory roles of this signaling pathway have been reported in other studies [32, 33]. Additionally, we observed that in mouse OC, IPI‐549 reduced the expression of PD‐L1. Similar results have been previously reported in multiple cancers, in which inhibitors of various components of the PI3K‐AKT‐mTOR pathway have been shown to suppress PD‐L1 expression [34, 35]. Further exploration of the mechanisms involved is needed.

Our in vivo and ex vivo experiments revealed that a PI3Kγ inhibitor remodeled the TIME of OC by reprogramming TAMs and improved the sensitivity of OC cells to anti‐PD‐1 therapy, providing theoretical evidence for a new combination of a PI3Kγ inhibitor combined with a PD‐1/PD‐L1 inhibitor for clinical use to improve OC immunotherapy efficacy. While the addition of IPI‐549 enhanced the response to anti‐PD‐1 therapy in mice, this effect may not be directly translatable to humans. The in vivo experiments conducted in this study did not reveal any drug toxicity associated with the PI3Kγ inhibitor. However, it is essential to perform toxicity testing at various drug concentrations, considering individual patient differences such as age, body surface area, and underlying diseases, prior to initiating clinical trials in OC. Importantly, the above results have only been based on an initial investigation of the mechanism in the laboratory, and more in‐depth studies are needed before the findings can be applied in the clinic to provide new directions for the treatment of patients with recurrent or refractory OC.

## 4 Conclusions

In conclusion, this study revealed that OC is an immunosuppressive tumor in which M2 macrophages predominate throughout disease progression and that PI3Kγ and PD‐L1 are closely associated with the development and prognosis of OC. The PI3Kγ inhibitor used in this study has no antitumor effect on OC cells when used alone; however, its combination with anti‐PD‐1 enhances the antitumor effects of anti‐PD‐1 therapy on these cells. Our preliminary investigation into the underlying mechanism revealed that this PI3Kγ inhibitor can augment antitumor effects by modulating the PI3Kγ‐AKT‐NF‐κB pathway, thereby remodeling the TIME of OC and transitioning it to an immunoactivated state. This modulation increases the sensitivity of OC cells to anti‐PD‐1 treatment.

## 5 Materials and Methods

### 5.1 Data Acquisition and Processing

Gene expression data and clinical data for OC patients were downloaded from the TCGA (https://www.cancer.gov/ccg/research/genome‐sequencing/tcga) and the Genotype‐Tissue Expression (GTEx, gtexportal.org/home/) databases. The GSE32062 dataset and the expression matrix and metadata‐information file of the single‐cell RNA‐sequencing dataset GSE154600 were retrieved from the Gene Expression Omnibus (GEO, https://www.ncbi.nlm.nih.gov/geo/) database. The CIBERSORT algorithm was used to perform Pearson correlation analysis of the gene expression of PIK3CG and immune infiltrating cells. Hallmark pathway and Kyoto Encyclopedia of Genes and Genomes (KEGG) pathway analyses were performed on specific genes. |log2‐fold change (FC)| ≥ 1.5 and *p* < 0.05 were considered statistically significant. The expression of the PIK3CG protein in OC was detected by querying the Human Protein Atlas (HPA, http://www.proteinatlas.org/) database for detection. The data were subjected to standardized preprocessing and log transformation using appropriate R packages. Single‐cell sequencing data were analyzed via the “Seurat” R package according to methods described in previous studies [36]. The analysis mainly included constructing objects, data standardization, data dimensionality reduction clustering, and identifying marker genes. All of the data are available for free online.

### 5.2 Clinical Tissue Sample Collection

Patients who underwent cytoreductive surgery for HGSOC from 2009 to 2018 in our hospital were enrolled in this study. Patients with the following characteristics were excluded: non‐HGSOC, without complete paired paraffin tissue specimens, or with malignant tumors of other organs. In accordance with the inclusion and exclusion criteria, paired paraffin‐embedded specimens of primary‐metastatic‐recurrent HGSOC lesions and paired paraffin‐embedded specimens of lesions and healthy ovaries from HGSOC patients (stage I) were collected.

### 5.3 In Vitro Cell Coculture System and Drug Intervention

The detailed methods used for IHC, hematoxylin and eosin (HE), cell culture, PBMCs/MSLs isolation, and treatment are described in the Supporting Information. Human OC lines (A2780, SKOV‐3, and OVCAR‐8) were cocultured with PBMCs, and ID8 cells were cocultured with MSLs. In accordance with published methods [25], after induction of differentiation, PBMCs/MSLs were seeded in a 6‐well plate with 1×10^6^ cells per well in the lower chamber. An insert with 0.4 µm pores (Cell Culture Insert, #3450, Corning, USA) was placed in each well, and 5×10^5^ OC cells were seeded with 1.5 mL of fresh growth media in the upper chamber (Figure S1A). The cells in coculture were incubated at 37°C and 5% CO_2_ for 24 h or 48 h and then divided into groups to observe the effects of IPI‐549 (a PI3Kγ inhibitor, eganelisib, Selleck, USA) [37] and anti‐PD‐1 (nivolumab, Selleck, USA) alone or in combination with OC in a cell coculture system. In addition, the control and positive control groups were treated with DMSO and PTX, respectively. The cell experiments, including FCM, cell proliferation, migration, invasion capacity and apoptosis assays, ELISA, and Western blotting, are described in the Supporting Information.

### 5.4 Mice and OC Models

C57BL/6J female mice (6 weeks) were obtained from GemPharmatech Co., Ltd. (Jiangsu, China). A total of 1×10^7^ or 5×10^6^ ID8 cells were subcutaneously or intraperitoneally (i.p.) injected into the mice, respectively. The body weights and subcutaneous tumor sizes of the mice were monitored every three days to assess tumor progression. Tumor volume = (length×width^2^)/2. The intraperitoneal tumor burden was measured via IVIS lumina III in vivo imaging (PerkinElmer, USA). The mice were randomized into five groups when the subcutaneous tumors were approximately 0.5 × 0.5 cm in size. In accordance with published methods for administration and dosage [16, 38, 39], for the positive control group, 20 mg/kg PTX (Sigma‒Aldrich, USA) was injected into (i.p.) the mice every three days. IPI‐549 was dissolved in sodium carboxymethyl cellulose (CMC‐Na) and administered by gavage to the mice every day at 30 mg/kg, and 250 µg/mouse anti‐mouse PD‐1 (CD279, clone RMP1‐14, BioXcell, USA) was administered via i.p. every three days. Controls were treated with the same volume of normal saline and CMC‐Na via i.p. and gavage, respectively (Figure S1B). The subcutaneous tumor model mice were sacrificed after one month of drug administration. Some tissues were frozen in liquid nitrogen for Western blotting, and the other tissues were fixed and embedded for IHC. In the intraperitoneal tumor model mice, drug administration was started 24 h after tumor implantation, with each dose administered as described above for a total of 4 doses of i.p. injection and 11 doses of gavage. The mice were injected with D‐luciferin potassium salt and imaged with an IVIS Lumina III system. Exfoliated cells and supernatants were obtained from the peritoneal cavities of the mice for FCM and ELISA, respectively. A second group of mice received the same treatment to track their survival rate.

### 5.5 Statistical Analyses

Statistical analysis was performed via IBM SPSS 22.0 software (SPSS Inc., Chicago, IL, USA) and R packages. Graphs were generated using GraphPad Prism v8, Adobe Illustrator CC 2019 software, and BioRender.com. In all figures, the data points and bar graphs represent the means of independent biological replicates. The results are presented as the means ± SDs. Comparisons between two groups were performed using an unpaired or paired two‐sided Student's *t*‐test; comparisons of multiple conditions were performed via one‐way analysis of variance. Survival curves were plotted using the Kaplan‒Meier method, and statistical probabilities were generated using the log‐rank test. *p‐*Values less than 0.05 were considered statistically significant (*, *p* < 0.05; **, *p* < 0.01; ***, *p* < 0.001).

## Author Contributions

**C.J**. finished and statistically analyzed the experiments and wrote the manuscript. **R.L**. assisted in completing the in vivo experiments. **Z.L**. provided practical suggestions and critically revised the manuscript. All the authors have read and approved the final manuscript.

## Conflicts of Interest

The authors declare no conflicts of interest.

## Ethics Statement

This study was approved by the Ethics Committee of West China Second University Hospital of Sichuan University (protocol number 2021–207). Written informed consent for retrospective data review and specimen use was obtained from all the subjects who participated in the study. Animal experiments were conducted in accordance with the West China Second University Hospital of Sichuan University Animal Care and Use Committee guidelines (protocol number 2021/057‐2).

## Supporting information



Supporting Information

## Data Availability

All data are available in the main text or the Supporting Information.
