# Supplementary material for: A Phosphatidylinositol 3‐Kinase Gamma Inhibitor Enhances Anti‐Programmed Death‐1/Programmed Death Ligand‐1 Antitumor Effects by Remodeling the Tumor Immune Microenvironment of Ovarian Cancer
Source: MedComm (2020). 2025 Jul 23;6(8):e70223. doi: 10.1002/mco2.70223 (PMC12284418; doi:10.1002/mco2.70223)
Supplement: Supplementary file 1 — Supporting Information [file MCO2-6-e70223-s001.docx]

Supplementary methods

**Immunohistochemistry (IHC), multiplex immunohistochemistry (mIHC), haematoxylin and eosin (HE) staining**

IHC, mIHC and HE staining were performed on formalin-fixed, paraffin-embedded tissue samples according to the manufacturer’s instructions. Changes in the expression of PI3Kγ (Abcam, ab154598), PD-1 (CST, 86163T), PD-L1 (CST, 13684T), CD68 (Abcam, ab955), CD86 (Abcam, ab234401) and CD163 (Abcam, ab316218) during HGSOC disease progression were detected by IHC and mIHC. In addition, PD-L1 (CST, #64988) in tumour tissues from mice with subcutaneous ovarian cancer was measured via IHC. Finally, images were acquired using a microscope (DP73 Olympus, Japan) and a digital slide scanner (Pannoramic MIDI, 3DHISTECH, Hungary). PI3Kγ, CD86 and CD163 expression was assessed using a scoring system on the basis of cell staining intensity and positive cell range, PD-1 expression was scored on the basis of the number of positive cells, and PD-L1 expression was scored on the basis of the combined positive score (CPS) and the tumour cell proportion score (TPS) method as previously described^1^. Investigator bias was avoided by two investigators independently scoring coded sections.

**PBMC/MSL isolation and treatments**

Human PBMCs were isolated from healthy donors following density gradient centrifugation (FicollPlus 1.077, Solarbio, China) at room temperature at 800 × g for 25 min. MSLs were acquired from healthy female mice (C57BL/6J, 12–16 weeks). In brief, the mouse spleen was removed, washed with 2 mL of phosphate-buffered saline (PBS), minced into 2–4 mm pieces and finely ground. A single-cell suspension was then obtained by passing the mixture through a 70 μm cell mesh and then centrifuged in the same way as above to obtain the MSL. After isolation, the cells were washed twice in PBS and cultured in a Rosewell Park Memorial Institute (RPMI) 1640 medium (HyClone, USA) supplemented with 10% foetal bovine serum (FBS) (Gibco, USA) and 1% penicillin‒streptomycin (Beyotime, China) in a 5% CO2 incubator at 37 °C. To induce differentiation into mature cells, PMBCs or MSLs were stimulated with M-CSF (10 ng/mL, PEPROTECH, USA) for 7 days and with IL-4 or IL-13 (20 ng/mL, PEPROTECH, USA) for 24 h^2^.

**Cell culture**

The human ovarian cancer cell lines A2780, SKOV-3, OVCAR-8, and the mouse (Luc) ID8 cell line were kindly donated by the Laboratory of Gynaecologic Oncology Biotherapy, West Maternal and Child Research Institute, and all the cell lines had STR identification certificates. A2780, SKOV-3, OVCAR-8, and (Luc) ID8 cells were grown in RPMI 1640 medium supplemented with 10% FBS in a 5% CO_2_ incubator at 37 °C.

**Flow cytometry (FCM)**

The prepared cells were first incubated with TruStain FcX™ (anti-mouse CD16/32) (BioLegend, USA) to block nonspecific binding of immunoglobulin to the Fc receptors and then stained with surface marker-specific antibodies at 4 °C for 20 min. Isotype-matched IgG was used as a negative control for each immunostaining procedure. The following fluorescent antibodies were purchased from BioLegend: PBMC panel antibody reagents: PE/Cyanine7 anti-human CD11b, Brilliant Violet 421™ anti-human CD68, APC anti-human CD86, PE anti-human CD206 (MMR), FITC anti-human CD3, Brilliant Violet 510™ anti-human CD8, Brilliant Violet 650™ anti-human CD33, and APC/Cyanine7 anti-human HLA-DR. Exfoliated cells from the peritoneal cavity of the mouse were obtained by centrifuging the mixture and then resuspending in PBS. Exfoliated cell/MSL panel antibody reagents: APC/Fire™ 750 anti-mouse/human CD11b, FITC anti-mouse F4/80, Brilliant Violet 421™ anti-mouse I-A/I-E, APC anti-mouse CD163, Brilliant Violet 605™ anti-mouse CD3, PE/Cyanine7 anti-mouse CD8a, Brilliant Violet 510™ anti-mouse Ly-6G, PE anti-mouse Ly-6C, and Brilliant Violet 785™ anti-mouse CD274. 7-AAD viability staining solution (BioLegend, USA) was also used for live/dead cell discrimination. Data were acquired using a BD Celesta and analysed using FlowJo v10. All experiments were performed in triplicate in three independent experiments.

**Cell proliferation assay**

The proliferation of ovarian cancer cells after coculture with PBMCs/MSL and treatment according to group was evaluated using a Cell Counting Kit-8 (CCK-8) assay. Briefly, in 96-well plates, ovarian cancer cells (2×10^3^/well) were seeded, and 10 μL of CCK-8 reagent (Biosharp, China) was added directly to the culture wells. The cells were then incubated at 37 °C for 90–120 min. The absorbance was measured using a microplate reader (Thermo Fisher, USA) at a wavelength of 450 nm.

**Cell** **migration and invasion capacity**

Cell migration was assayed using inserts with 8.0 µm polycarbonate membranes in 24-well plates (#3422, Corning, USA). After coculture with PBMCs/MSL and treatment according to the groups, 6×10^4^ ovarian cancer cells were resuspended in 200 μL of serum-free medium and plated into each upper chamber. In addition, 600 μL of medium supplemented with 20% FBS was added to the bottom of each well as a chemoattractant. Nonmigrating cells were removed after a period of incubation. Transwell membranes were fixed with 4% paraformaldehyde and stained with 0.5% crystal violet. Similar experimental procedures for cell invasion were carried out using Matrigel-coated invasion chambers from Corning. To count the fixed cells, five random fields of vision were captured and counted using a microscope and ImageJ software.

**Cell apoptosis**

After coculture with PBMCs/MSLs and treatment according to groups, the ovarian cancer cells were collected and resuspended in PBS. The cells were stained with an Annexin V-FITC/PI Apoptosis Assays Kit (KeyGEN BioTECH, China) according to the manufacturer’s protocols. The percentage of apoptotic cells was assessed with a Beckman FC500 and analysed using FlowJo v10.

**Enzyme-linked immunosorbent** **assay (ELISA)**

After ovarian cancer cells were cocultured with PBMCs/MSLs and treated according to the planned groups, the supernatant was collected for further analysis. Following the manufacturer’s protocols, ELISA was used to detect the proinflammatory cytokines IL-12 and TNF-α and the anti-inflammatory cytokines IL-10 and TGF-β.

**Western blot**

The total protein extracted from each cell line or tissue sample was dissolved in RIPA buffer, and the lysates were centrifuged at 12,000 rpm for 15 min at 4 °C. The supernatants were collected, and the protein concentration was quantified via a BCA protein assay. The cell lysates were subjected to SDS‒PAGE and transferred to PVDF membranes, followed by immunoblotting with primary antibodies, including an Akt (pan) 1/2/3 antibody, a phospho-Akt (thr308) antibody, a pNF-κB (S536) antibody, a CEBPβ antibody (Abways, China), a pCEBPβ antibody (CST, USA) and a PI3 kinase p110 gamma (2D10) mouse mAb (ZENBIO, China). The goat anti-rabbit IgG (H+L) HRP and goat anti-mouse IgG (H+L) HRP secondary antibodies were obtained from Abways (China). GAPDH was used as a control.

**Table S1** Clinicopathological characteristics of patients with paired primary, metastatic, and recurrent HGSOC lesions (n=10).

| Clinicopathological characteristics | Median (range)/Cases (percentage) |
| --- | --- |
| Age (years)^*^  FIGO stage  ⅢC  Ⅳ  Surgery timing  PDS  NAC+IDS  Residual disease (PDS/IDS)  ≤1  >1  Number of platinum-based chemotherapy after the initial operation  PFS (months)  CA125 at relapse (U/mL)  Platinum-based chemotherapy before SCS  Yes  No  Residual disease(SCS)  ≤1  >1 | 49.50 (41, 61)  10 (100%)  0 (0%)  6 (60%)  4 (40%)  10 (100%)  0 (0%)  8 (3, 16)  14.50 (6, 64)  104.60 (56.40, 4167.80)  5 (50%)  5 (50%)  7 (70%)  3 (30%) |

Annotation: HGSOC, high-grade serous ovarian cancer; PDS, primary debulking surgery; NAC, neoadjuvant chemotherapy; IDS, interval debulking surgery; PFS, progression-free survival; SCS, secondary cytoreductive surgery; * is the age of the patient at the time of initial diagnosis of the tumour.

**
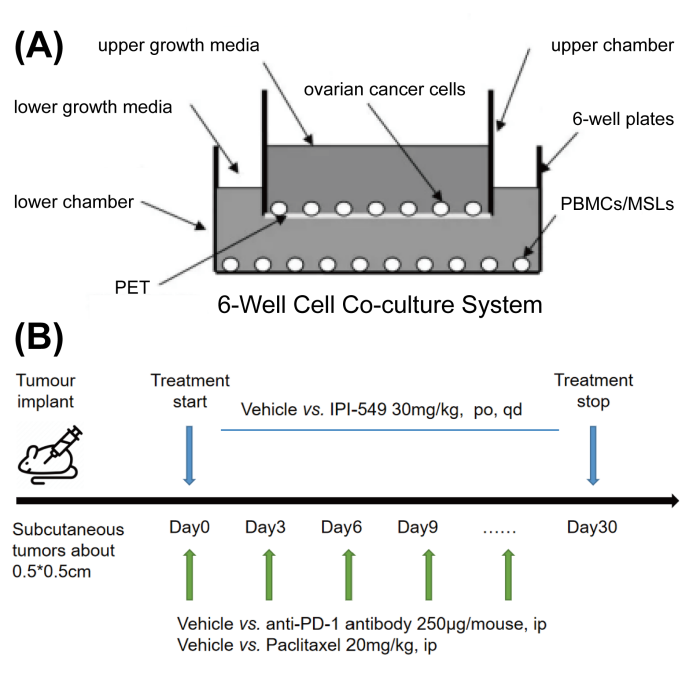
**

**Figure S1** **(A)** Diagram of the cell coculture system. **(B)** Methods of intervention and treatment.


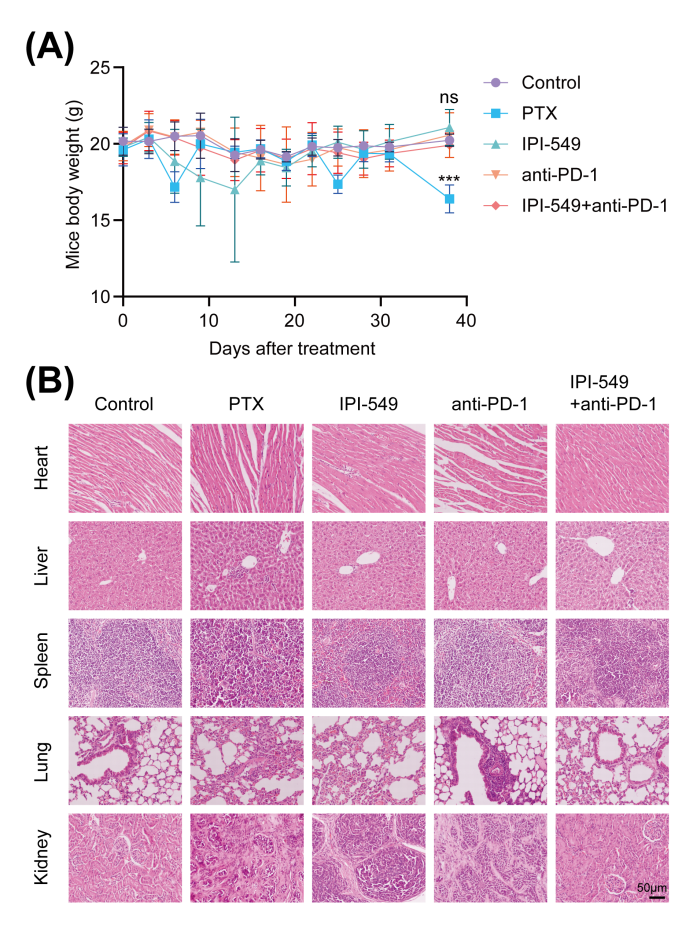


Figure S2 Effects of IPI-549 and anti-PD-1 agents alone or in combination on body weight and organ toxicity in mice. (A) Body weights of the mice. (B) HE staining of sections of the mouse heart, liver, spleen, lung and kidney. 200×; scale bar: 50 μm. ****P* < 0.001.

**References:**

1. Doroshow DB, Bhalla S, Beasley MB, et al. PD-L1 as a biomarker of response to immune-checkpoint inhibitors. *Nature Reviews Clinical Oncology*. 2021;18(6):345-362.

2. Nielsen MC, Andersen MN, Møller HJ. Monocyte isolation techniques significantly impact the phenotype of both isolated monocytes and derived macrophages in vitro. *Immunology*. 2019;159(1):63-74.
